# Extended-Spectrum β-Lactamases (ESBL): Challenges and Opportunities

**DOI:** 10.3390/biomedicines11112937

**Published:** 2023-10-30

**Authors:** Asmaul Husna, Md. Masudur Rahman, A. T. M. Badruzzaman, Mahmudul Hasan Sikder, Mohammad Rafiqul Islam, Md. Tanvir Rahman, Jahangir Alam, Hossam M. Ashour

**Affiliations:** 1 Department of Pathology, Faculty of Veterinary, Animal and Biomedical Sciences, Sylhet Agricultural University, Sylhet 3100, Bangladesh; 2 National Institute of Infectious Diseases and Vaccinology, National Health Research Institutes, Zhunan Town 350, Miaoli County, Taiwan; 3 ABEx Bio-Research Center, East Azampur, Dhaka 1230, Bangladesh; 4 Department of Pharmacology, Faculty of Veterinary Science, Bangladesh Agricultural University, Mymensingh 2202, Bangladesh; 5 Livestock Division, Bangladesh Agricultural Research Council, Farmgate, Dhaka 1215, Bangladesh; 6 Department of Microbiology and Hygiene, Faculty of Veterinary Science, Bangladesh Agricultural University, Mymensingh 2202, Bangladesh; 7 Animal Biotechnology Division, National Institute of Biotechnology, Dhaka 1349, Bangladesh; 8 Department of Integrative Biology, College of Arts and Sciences, University of South Florida, St. Petersburg, FL 33701, USA

**Keywords:** ESBL, combination therapy, antibiotics, resistance

## Abstract

The rise of antimicrobial resistance, particularly from extended-spectrum β-lactamase producing *Enterobacteriaceae* (ESBL-E), poses a significant global health challenge as it frequently causes the failure of empirical antibiotic therapy, leading to morbidity and mortality. The *E. coli*- and *K. pneumoniae*-derived CTX-M genotype is one of the major types of ESBL. Mobile genetic elements (MGEs) are involved in spreading ESBL genes among the bacterial population. Due to the rapidly evolving nature of ESBL-E, there is a lack of specific standard examination methods. Carbapenem has been considered the drug of first choice against ESBL-E. However, carbapenem-sparing strategies and alternative treatment options are needed due to the emergence of carbapenem resistance. In South Asian countries, the irrational use of antibiotics might have played a significant role in aggravating the problem of ESBL-induced AMR. Superbugs showing resistance to last-resort antibiotics carbapenem and colistin have been reported in South Asian regions, indicating a future bleak picture if no urgent action is taken. To counteract the crisis, we need rapid diagnostic tools along with efficient treatment options. Detailed studies on ESBL and the implementation of the One Health approach including systematic surveillance across the public and animal health sectors are strongly recommended. This review provides an overview of the background, associated risk factors, transmission, and therapy of ESBL with a focus on the current situation and future threat in the developing countries of the South Asian region and beyond.

## 1. Introduction

Antibiotics are the first drugs of choice to treat infectious diseases. A rise in infectious diseases, increasing rate of drug resistance, and indiscriminate use of antibiotics are the reasons behind the high usage of antibiotics in developing countries. In recent years, the Asia-Pacific region had a significant share of the global antibiotic market, a market that is expected to be valued at USD 59.72 billion by the year 2028 [1].

Antimicrobial resistance (AMR) has a negative impact on achieving Sustainable Development Goals (SDG), food safety, and food security. In the antimicrobial resistance (AMR) era, the evolving resistance caused by extended-spectrum β-lactamases (ESBLs) led to higher morbidity, prolonged hospital stays, and expensive treatment options [2]. ESBLs are Gram-negative bacteria of the *Enterobacteriaceae* family that carry ESBL genes in their plasmids or chromosomes, produce β-lactam hydrolyzing enzymes, and are rightly considered to be among the most challenging pathogens by the World Health Organization (WHO). ESBL-producing *Enterobacteriaceae* (ESBL-E) confer resistance to penicillin—in addition to aztreonam and first-, second-, and third-generation cephalosporins—but are unable to hydrolyze cephamycin or carbapenems [3]. Carbapenem has been the drug of first choice for treating ESBL-E-induced infection for a long time [4]. This is changing, though, due to many factors including the recent emergence of carbapenemase-producing bacteria. Thus, there is an urgent need to develop alternative approaches.

It is well known that the misuse or overuse of antibiotics in both human and animal populations is responsible for the evolution of drug-resistant bacteria via gene mutations or horizontal transmission of resistance genes by plasmids [5]. ESBL-E are commensal bacteria in both humans and animals and can be a major threat to food safety and food security. Commensal ESBL reservoirs in the environment have experienced recent dramatic increases due to the co-transmission of ESBL-E between the human and animal populations, which can occur through several direct and indirect routes of transmission. Pathogenic bacteria in the environment are able to acquire ESBL genes from commensal bacteria and can pose significant health risks to humans and animals [6].

It is estimated that over 1.5 billion people are colonized with ESBL-E, including a majority in developing countries [7]. Moreover, reports from South Asian developing countries, including Bangladesh, India, and Pakistan, indicated a high prevalence of ESBL-E and other multidrug-resistant (MDR) superbugs [8]. The increased dissemination of ESBL-E in humans and animals in different areas of the globe has led to the current resistance situation. More studies on ESBL surveillance in humans and animals need to be conducted. The One Health approach is a promising approach to try to tackle the escalating issue of ESBL-E resistance. This review presents a comprehensive insight into ESBL. It covers co-transmission routes between humans and animals as well as updated diagnostic and treatment strategies. It covers the current status, potential future threats, and opportunities to intervene. While recognized as a global problem, examples from developing countries in South Asia are provided.

## 2. Extended-Spectrum β-Lactamases (ESBL) and ESBL Producers

ESBL are hydrolyzing enzymes secreted by several Gram-negative bacteria of the family Enterobacteriaceae. They cause the inactivation of broad-spectrum oxyimino-cephalosporin (third- and fourth-generation) and monobactam (aztreonam) but not cephamycin (cefoxitin) or carbapenems (meropenem, imipenem, ertapenem, and doripenem) [9,10]. Generally, these enzymes are neutralized by β-lactamase inhibitors (BLIs) such as clavulanic acid, sulbactam, and tazobactam [9]. Genes that encode ESBL are mostly found on transposons or insertion sequences of plasmids in association with other resistance genes. As a result, they can spread rapidly, causing resistance to multiple antimicrobials such as aminoglycosides, trimethoprim, sulphonamides, tetracyclines chloramphenicol, and fluoroquinolone [11,12,13].

ESBL are produced by the nosocomial pathogens *E. coli*, *Klebsiella pneumoniae*, *Acinetobacter baumannii*, *Pseudomonas aeruginosa*, and *Enterobacter* spp. [14]. Among a wide range of Gram-negative bacterial species of different families harboring ESBL genes, *E. coli* is the most common host, followed by *K. pneumoniae*. Among the different variants of ESBL-producing *E. coli*, the ST131 clone is the most dominant [3].

The ESBL-encoding genes are highly diverse in nature and can be classified into many families with unique characteristics such as *bla*TEM, *bla*SHV, and *bla*CTX-M. TEM 1, the first plasmid and transposon-mediated β-lactamase, was isolated from the blood culture of a named Temoniera in Greece in the early 1960s [15]. It has spread worldwide and is now found in many species of the family *Enterobacteriaceae*, *P. aeruginosa*, *Hemophilus influenzae*, and *Neisseria gonorrhoeae* [16]. The SHV-1 type is common in *Klebsiella* spp. and *E. coli* [16]. CTX-M-type ESBL are predominant in *E. coli*, *K. pneumoniae*, *S. enterica* serovar *Typhimurium*, and *Shigella* spp. [17]. The plasmid-mediated OXA and AmpC-type ESBL were discovered in *P. aeruginosa* and *K. pneumoniae* isolates, respectively [16,18]. A series of *Salmonella* serovars, including *S. enteritidis*, *S. newport*, and *S. paratyphi*, have been characterized as ESBL producers that have been linked to serious foodborne gastroenteritis in humans [19].

## 3. Classification and Evolution of ESBL

**Figure 1:**
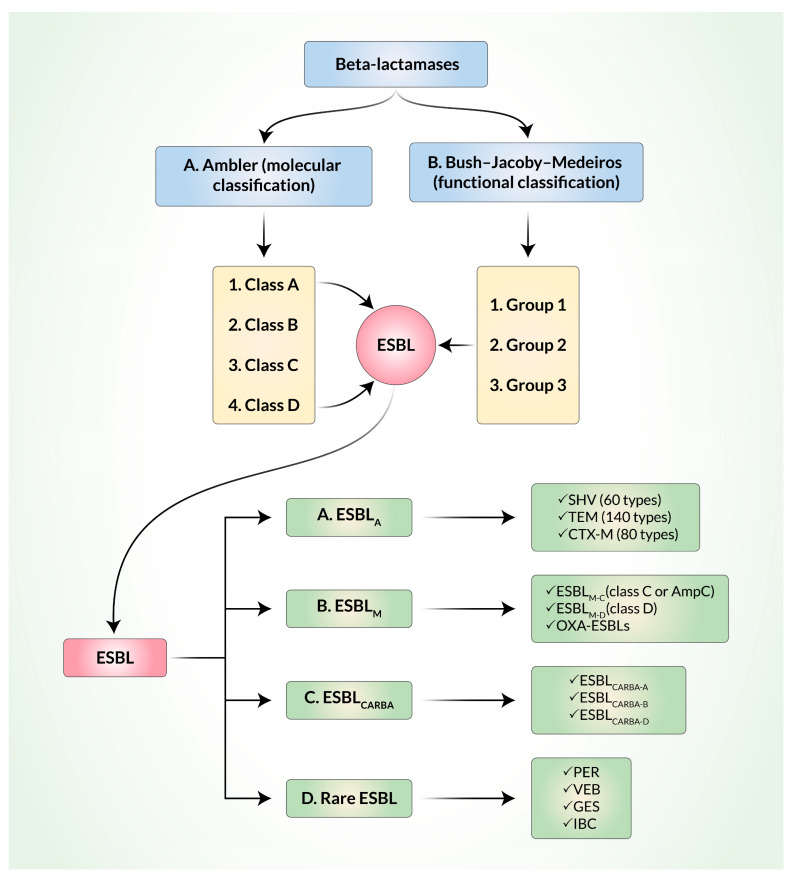
Classification of extended-spectrum β-lactamases (ESBL). A. Ambler (molecular) classification. B. Bush–Jacoby–Medeiros classification.

ESBL are structurally and functionally mutated versions of β-lactamases. It is noteworthy that β-lactamases can be defined and classified by the Ambler classification system on the basis of molecular structure [20] and by the Bush–Jacoby–Medeiros classification system on the basis of function (Figure 1). Among the four classes (A, B, C, and D) of the Ambler classification, ESBL belong to classes A and D where serine is used as an enzyme active center. According to the Bush–Jacoby–Medeiros system, β-lactamases are classified into groups 1 to 3, along with several subgroups, on the basis of lysis of β-lactam substrates and the effects of inhibitors. Ambler’s A and D classes of ESBL belong to group 2 in the Bush–Jacoby–Medeiros system. In order to keep track of the newly evolved β-lactamases, Bush and Jacoby later proposed an update to the original Bush–Jacoby–Medeiros functional classification system of β-lactamases [11]. In both the original version and the updated 2009 version of the classification, ESBL belonged to group 2.

More recently, ESBL have been classified into three main groups: Ambler class A ESBL (ESBL_A_), miscellaneous ESBL (ESBL_M_), and ESBL that degrade carbapenems (ESBL_CARBA_) [9]. Most ESBL in the world belong to the ESBL_A_ group, which includes several types of sulfhydryl reagent variable (SHV) β-lactamases, Temoniera (TEM) β-lactamases, and cefotaxime-M (CTX-M) β-lactamases [21]. About 90% of TEM-1 harboring *E. coli* can confer resistance to ampicillin, penicillin, and first-generation cephalosporins but not to oxyimino cephalosporin. Additionally, SHV-1 (68% similar to TEM on the basis of amino acid sequences) can provide resistance to penicillin, tigecycline, and piperacillin but not to oxyimino cephalosporin [22]. During the 1980s, evolution of SHV-1 and TEM-1 from non-ESBL to ESBL in *K. pneumoniae* and *E. coli* strains, respectively, via specific amino acid substitutions, made them more capable of hydrolyzing oxyimino-cephalosporins [13]. Among the 140 TEM and 60 SHV types identified, some are capable of inactivating third-generation cephalosporins and aztreonam [22].

More recent outbreaks involving ESBL have been mediated by the CTX-M type rather than the TEM type or the SHV type [23]. CTX-M-type ESBL (first reported in 1989 in Munich, Germany) preferentially hydrolyze cefotaxime over ceftazidime and are inhibited by tazobactam [24]. They are distinct from TEM-type and SHV-type ESBL. The ESBL enzyme-encoded *bla* genes originated from the chromosomes of *Kluyvera* spp. (non-pathogenic *Enterobacteriaceae*). CTX-M ESBL are grouped into six major types—CTX-M-1, CTX-M-2, CTX-M-8, CTX-M-9, CTX-M-25, and KLUC—on the basis of ≥10% variance in amino acid sequence identity and several minor variants within the groups [25].

More than 80 CTX-M types have been reported in both hospitals and communities as well as in food animals, fresh vegetables, water, and the environment [22]. Mobile genetic elements (MGEs) such as IS*Ecp1* and IS*CR1* play an important role in transferring *bla*CTX-M genes from the chromosomes of *Kluyvera* spp. into the plasmids of *E. coli*. The gene expression of *bla*CTX-M is enhanced by several active promoter sequences encoded in some MGEs, resulting in increased cephalosporin resistance in *E. coli* in hospital settings [26]. While CTX-M-type ESBL are mainly detected in plasmid incompatibility groups, chromosomal integration was also reported [25]. In humans, CTX-M-15 (CTX-M-1 group) and CTX-M-14 (CTX-M-9 group) are more prevalent, whereas CTX-M-1 (CTX-M-1 group) is more predominant in animals [27]. Other CTX-M groups were reported in specific regions, such as the CTX-M-2 and CTX-M-8 groups in South America and the CTX-M-2 group in Japan [25].

ESBL_M_ are further classified into ESBL_M-C_ (class C, plasmid-mediated AmpC) and ESBL_M-D_ (class D). The AmpC group confers resistance to penicillin, third- and fourth-generation cephalosporins, and, sometimes, to carbapenems. They are inhibited by cloxacillin and boronic acid. Some OXA-ESBL are also classified within the ESBL_M_ group. Carbapenem-resistant ESBL are also divided into ESBL_CARBA-A_, ESBL_CARBA-B_, and ESBL_CARBA-D_ [28]. ESBL_CARBA_ can degrade all β-lactam antibiotics. They are inhibited by either ethylenediaminetetraacetic acid (EDTA) or dipicolinic acid (DPA), as in the cases of Metallo- β-lactamases (MBLs), boronic acid, or avibactam. Some OXA enzymes are also included in the ESBL_CARBA_ group. OXA-type β-lactamases that belong to Ambler class D are different from TEM and SHV, have the ability to hydrolyze oxacillin and cloxacillin, and are not inhibited by clavulanate acid. They have been mainly detected in *P. aeruginosa* and a much lesser percentage (1–10%) have been detected in *E. coli*. Other rarely found ESBL that are transmitted through plasmids are Pseudomonas extended resistant (PER), Vietnam ESBL (VEB), Guiana extended-spectrum (GES), and integron-borne cephalosporinase (IBC) [3].

## 4. Mechanism of Resistance and Dissemination of Resistant Genes

**Figure 2:**
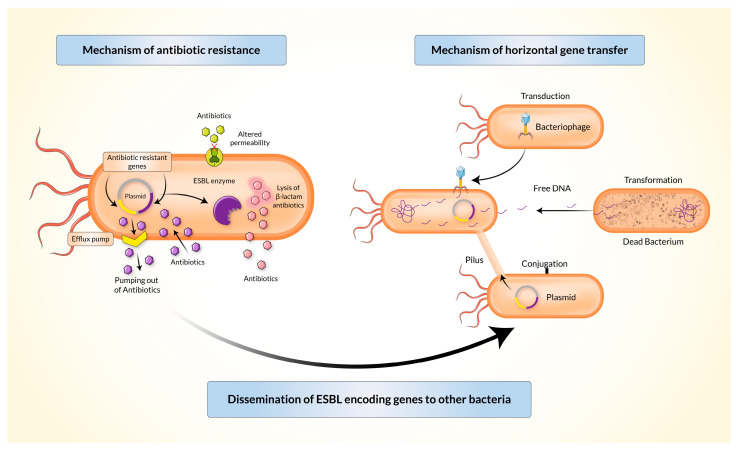
Mechanisms of antibiotic resistance and horizontal gene transfer by extended-spectrum β-lactamase producing *Enterobacteriaceae* (ESBL-E).

Gram-negative bacteria may inactivate β-lactam antibiotics (penicillin and cephalosporin) through several mechanisms (Figure 2). The periplasm of Gram-negative bacteria releases β-lactamase which has a higher affinity towards β-lactam antibiotics than the affinity of β-lactam antibiotics to their targets. The gene coding β-lactamase may be located in the immobile genetic chromosomes (in Enterobacter species) or extra-chromosomal MGEs such as a plasmid, integrin, or a transposon. The resistant genes evolve either gene-level mutations or acquisition of resistant genes from other bacteria of the same or different species.

Bacterial integrons, described at the end of the 1980s, act as a vehicle for the transmission (intraspecies or interspecies) of resistant genes by the acquisition of sequences present in transposons and/or conjugative plasmids through the process of horizontal gene transfer [29]. This can happen through transformation, transduction, or conjugation (Figure 2). Genes encoding TEM-type β-lactamases are mostly carried and disseminated by Tn1, Tn2, or Tn3-like transposons. Genes encoding SHV-type β-lactamases can be mediated by both chromosomes and plasmids. Conjugative transmission is most commonly observed in the CTX-M type [3]. Five classes of integrons (*intI1*, *intI2*, *intI3*, *intI4*, and *intI5*) were found to play major roles in the dissemination of antibiotic-resistance genes [30].

Inhibitors used to block ESBL enzymes can help prevent the inactivation of β-lactam antibiotics. It is important to note that some β-lactamases may not be inactivated by some classical inhibitors such as clavulanate acid, sulbactam, and tazobactam [31,32]. Mechanisms of resistance in Gram-negative bacteria may also involve reduced membrane permeability through genomic mutations, decreased amounts of β-lactam antibiotics that can enter the cell, and a marked increase in antibiotic reflux from the periplasm to the exterior of the cell [31].

## 5. Diagnostic Tools for Detection of ESBL

Routine screening along with rapid detection of ESBL-producing bacteria in laboratory and hospital settings is essential in the therapeutic approach and infection control to suppress any outbreaks. The Clinical and Laboratory Standards Institute (CLSI) recommends a two-step process for the detection of ESBL [33]. The second part is only undertaken if the first step leads to a positive result. The first step involves a preliminary screening to detect sensitivity against some commonly used antibiotics such as cefotaxime, ceftriaxone, ceftazidime, or aztreonam. The second involves one of the available confirmatory tests to identify ESBL-producing organisms in the presence of β-lactamase inhibitor [34]. Tests recommended by CLSI for the screening of ESBL include Kirby–Bauer disks and Vitek (sensitivity 92–93%). The confirmatory tests may be performed using a double-disk synergy test (DDST), combination disk method, or E-test ESBL strips. The combination disk method has a very high sensitivity (100%) for testing cefotaxime and cefepime, whereas the E-test has a comparatively lower sensitivity for testing cefotaxime and ceftazidime (71–73%) or cefepime (90%) [22]. The phenotypic confirmatory method, double-disc synergy test, and E-test ESBL strip tests are easy to use in a laboratory setting, although none of these methods alone can identify all types of ESBL [32]. It is worth mentioning that there are also guidelines set by the European Committee on Antimicrobial Susceptibility Test (EUCAST) for the detection of ESBL [35].

In addition to phenotypic confirmatory tests, genotypic confirmatory tests are performed to identify certain enzymes and their variants released by ESBL producers through methods that include polymerase chain reaction (PCR) and nucleotide sequencing [22]. Other methods that can be used include the broth dilution method (BDM) [36], isoelectric point determination, DNA probes, the oligotyping method, PCR with restriction fragment length polymorphism analysis (PCR-RFLP), PCR with single-strand conformational polymorphism analysis (PCR-SSCP), and real-time-PCR [32]. The Cica Beta Test 1/HMRZ-86/Chromogenic cephalosporin is a rapid kit test (generates results within 15 min) that is used for detecting ESBL in Gram-negative rods from primary culture [37]. Matrix-assisted laser desorption ionization-time of flight mass spectrometry (MALDI-TOF) is another diagnostic tool that has been successfully used to detect ESBL [38]. Recently, the NG-Test CTX-M MULTI, a rapid immunochromatography technique (lateral flow), has proven to be useful for the detection of CTX-M-type enzymes (groups 1, 2, 8, 9, and 25), followed by the rapid identification of *Enterobacterales* in blood or urine samples using MALDI-TOF MS and flow cytometry [39,40]. Moreover, for the detection of SHV-positive *K. pneumoniae*, PCR with CRISPR-LbCas12a has demonstrated excellent sensitivity and specificity, and it is recommended for use in a hospital setting as it provides results in about two hours [41].

The applicability of these detection methods in different situations can have limitations due to the frequent mutations that lead to changes in patterns of ESBL subtypes. This can make diagnosis more complex and difficult. Different types of ESBL detection methods are summarized in Table 1.

## 6. Risk Factors and Mode of Transmission of ESBL-Producing Bacteria

Throughout the recent decades, ESBL-producing bacteria have been increasingly detected in hospital and community settings and have thus emerged as a serious health problem for humans and animals [42,43]. Reduced treatment options, complex infections, high mortality, and costly treatments are some of the major concerns for people infected with ESBL-producing organisms [2]. In the intensive care unit (ICU), ventilator-associated pneumonia by ESBL-producing bacteria has been detected in hospitalized patients [44]. In the human population, risk factors for hospital-borne colonization and infection with ESBL producers include prolonged hospital stay, use of hemodialysis, and intravascular catheters [45,46]. Community-borne infections may be related to many factors, including international traveling [47]. In veterinary medicine, cephalosporins are frequently used for the treatment of bacterial infections in farm animals and pet animals [48]. In South Asia, excessive use of over-the-counter (OTC) cephalosporins may be a major cause for increasing ESBL-producing bacteria in the animal population, which can further cocirculate in the human population via the food chain.

ESBL-producing enteric bacteria, such as *E. coli*, non-typhoidal *Salmonella* spp., and *Campylobacter* spp., are zoonotic pathogens spread to humans through the food chain and can transiently colonize the human gut. Resistant commensal *E. coli* acts as a vehicle to transmit genetic resistance determinants in the gut or via milk and meat. Resistant pathogenic *E. coli* may subsequently cause urinary tract infections in vulnerable patients [49]. In food-producing animals and pet animals, cephalosporin-resistant *E. coli* and *Salmonella* spp. cause high levels of mortality and morbidity which pose a risk of spread to humans via improper handling and inadequate cooking of food [50]. CTX-M-14 is predominant in Asian countries and has been detected in humans, pets, and poultry [19]. The CTX-M-15-producing human ST15 and ST101 *K. pneumoniae* clones have been reported to be widely disseminated in pets and horses [51]. The *bla*CTX-M-1 encoding IncI1 plasmids were commonly identified in *E. coli* isolates from animals and humans along with various sequence types (STs) of *E. coli* [52].

In addition to causing intestinal and urinary tract infections, ESBL-producing Gram-negative bacteria, such as *E. coli*, *Proteus* spp., *Pseudomonas aeruginosa*, and *Klebsiella* spp., can also be responsible for diabetic foot ulcers in individuals with underlying health conditions, potentially leading to amputation and death [53]. A high incidence of sternal wound infections caused by ESBL-producing *E. coli* has also been reported among patients in postoperative care after cardiac surgery [54].

**Figure 3:**
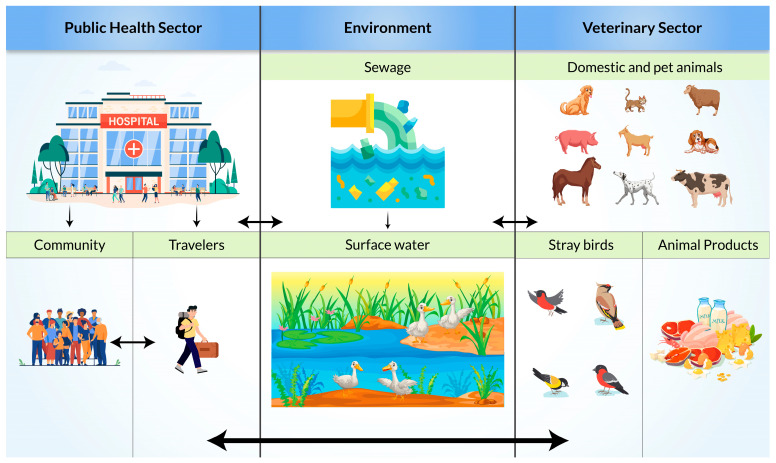
Possible transmission pathways of Extended-Spectrum β-Lactamase (ESBL)-producing bacteria.

Resistance transmission routes for ESBL-producing bacteria are complex (Figure 3). There are multiple direct and indirect transmission pathways from animal and inanimate sources to humans and from humans to animals and the environment [55]. Extended-spectrum β-lactamase-producing enterobacterales isolates were reported in farmers and livestock (pig and poultry) [56,57]. Lower genomic ESBL diversity was also seen in farming communities than in the general and clinical populations. This can indicate a higher possibility of the exchange of ESBL genes between reservoirs in farming communities through close contact. Additionally, molecular similarities between human and environmental reservoirs may be an indication of transmission from human wastewater to surface water [58]. Through the contaminated surface water, wild birds may get infected and act as vectors or even reservoirs for local dissemination [59]. A high prevalence in migratory birds (17% in Pakistan, 17.3% to 38.18% in Bangladesh) is an indication that migratory birds can be a potential carrier for transmission in Asian countries [60,61,62].

## 7. Possible Therapeutic Options

Resistance towards certain commonly prescribed antibiotics, such as penicillin and cephalosporins, can make these drugs ineffective for treating infections. Carbapenems have been considered the main therapeutic option for the treatment of ESBL-E [4]. The intravenous administration of carbapenem antibiotics is more efficient than its oral administration. However, injudicious overuse led to the emergence of carbapenem resistance.

Carbapenem-sparing strategies include the administration of non-carbapenem β-lactams (ceftolozane–tazobactam, ceftazidime–avibactam, temocillin, cephamycins, and cefepime) and non-β-lactams (aminoglycosides, quinolones, tigecycline, eravacycline, and fosfomycin).

For the non-carbapenem β-lactams, piperacillin–tazobactam (PTZ) combination is the most suitable alternative to carbapenems in the treatment of mild urinary tract infections (MIC ≤ 4 mg/L) [63,64]. Ceftolozane–tazobactam appears to be promising in the treatment of complicated intra-abdominal infections and complicated urinary tract infections [65]. Tazobactam and Avibactam are β-lactamase inhibitors but tazobactam is affected by the inoculum effect [63]. The effects of Tazobactam can be reduced by certain Gram-negative bacteria that are capable of releasing ESBL and AmpC beta-lactamases and can protect themselves through activation of efflux pumps and porin mutations. Avibactam has the ability to conserve the efficacy of ceftazidime against the highly prevalent β-lactamases, such as ESBL, and carbapenemases including OXA-48 and *K. pneumoniae* carbapenemase (KPC). Hence, the ceftazidime–avibactam combination produces better results for the majority of MDR Gram-negative bacteria [66]. Cephamycins include cefoxitin, cefotetan, moxalactam, cefmetazole, and flomoxef. Cephamycins are ineffective against AmpC cephalosporinases and porin mutations [67]. Cefepime, a fourth-generation cephalosporin that is less hydrolyzed by AmpC lactamases and ESBL than other cephalosporins, could help against low-risk infections (MIC ≤ 2 mg/L) [68]. However, there is a possible risk of mortality in some cases [43]. Temocillin (b-a-methoxy-derivative of ticarcillin), a new drug, has a narrow spectrum that is limited only to Enterobacterales and is not easily degraded by various β-lactamases [66].

For non-β-lactams, quinolones and aminoglycosides are good options. ESBL genes were shown to mediate quinolone resistance [69]. The spreading of aminoglycoside-modifying enzymes can impact microbial susceptibility to aminoglycosides [70]. Amikacin and the next-generation aminoglycoside plazomicin could be used for the treatment of urinary tract infections, including the treatment of acute pyelonephritis by plazomicin [71,72,73]. Tigecycline has efficacy against ESBL-producing *E. coli* and against multidrug-resistant (MDR) and extensively drug-resistant *Acinetobacter baumannii* and *K. pneumoniae* [66,74]. The tetracycline derivatives, Eravacycline and Omadacycline, have anti-ESBL activity that could be used to control Gram-negative bacteria [75,76]. Fosfomycin interferes with the synthesis of peptidoglycan by inhibiting phosphoenolpyruvate transferase and can be effective with urinary tract infections [66]. Fosfomycin is efficient for the treatment of acute uncomplicated cystitis [77]. Finally, monotherapy is generally less effective than combination therapy [78].

## 8. Current Status of ESBL in South Asian Developing Countries

In this era of antibiotic resistance, developing countries are considered as a hotbed for the spread of resistant bacteria due to the imprudent use of antibiotics, poor drug quality, lack of proper monitoring, as well as many other factors associated with individual and national poverty in many of these countries [6]. Bangladesh, India, and Pakistan are three densely populated South Asian developing countries that have high degrees of antimicrobial resistance in both the human and animal sectors. Availability of antibiotics over the counter, the tendency to self-medicate, lack of completion of antibiotic courses, unnecessary overprescribing of antibiotics by physicians, and the indiscriminate use of antibiotics in agriculture and veterinary practices are considered major causes of AMR in these countries [79,80].

ESBL have been frequently reported on the Asian subcontinent since the late 1990s. In Bangladesh, ESBL have been reported for more than two decades [81]. The globally dominant ESBL *bla*CTX-M-15 was first reported in India in the mid-1990s and is still a dominant ESBL type in India, Bangladesh, and Pakistan [82]. AMR surveillance in these countries is not comprehensive and there is a general underreporting of AMR. The largest proportions of these studies were conducted on humans. A significantly high proportion of AMR, MDR, and ESBL producers were detected in the period from 2015 to 2020 (Table 2 and Table 3).

In Bangladesh, a study in a tertiary care hospital in Dhaka revealed the presence of ~16% ESBL producers (15.75% *Escherichia coli*, 14.01% *Pseudomonas* spp., 36.84% *Proteus* spp., 18.57% *Klebsiella* spp., and 21.05% *Acinetobacter* spp.) in indoor (~50%) and outdoor (13%) patients [118]. A similar study has reported that 34% *of E. coli* isolated from extra-intestinal infection in patients were ESBL-producing [88]. Another study revealed a high prevalence of MDR ESBL-producing *E. coli* isolates in Bangladesh (most isolates were shown to have *bla*_CTX-M_), including the uropathogenic ESBL-producing *E. coli* clone O25:H4 [119]. Moreover, about 60% of ESBL-positive *E. coli* carrying *bla_CTX_*_-*M*-1_, *bla_CTX_*_-*M*-2_, *bla_CTX_*_-*M*-8_, *bla_CTX_*_-*M*-9_, *bla_CTX_*_-*M*-15_, *bla_CTX_*_-*M*-25_, *bla_TEM_*, and *bla_SHV_* genes were detected in human faecal sludge samples isolated from a Rohingya camps in Cox’s Bazar, Bangladesh [120]. Additionally, 74% ESBL-producing *E. coli* were detected in stool samples from healthy infants in rural areas of Bangladesh [62]. It is unknown whether the resistance was primarily acquired from the environment, vertically from the child’s mother, or through selective pressure from pediatric antibiotic overuse [121]. In a molecular study, CTX-M-type and SHV-type ESBL genes were detected in *E. coli*, *K. pneumoniae*, and *Enterobacter cloacae* isolated from surface water in Dhaka, Bangladesh [122]. The fairly common practice in rural areas of Bangladesh to dispose of infants’ stool in front yards or nearby ditches might have contributed to the transmission of resistant bacteria to domestic and stray birds and/or other animals [123]. It has been reported that crows act as potential carriers of human-pathogenic ESBL-producing *E. coli* ST13-O25b clones because of their foraging behaviors [124]. Household pigeon droppings were shown to contain *bla*CTX-M-15 genes of the ESBL-producing *E. coli* ST1408, known to be a bird-associated sequence [125]. Migratory birds traveling to Bangladesh have been reported to be a potential source of ESBL-producing *E. coli* carrying blaTEM, blaCTX-M, blaCMY, and blaSHV genes [126]. To alleviate the escalating food shortage for an increasing population in Bangladesh, antibiotics are overused to promote growth and to prevent and treat diseases in food animals. A high percentage of ampicillin-resistant *bla*TEM gene (91.25%) was reported in *E. coli* isolated from cloacal swabs of live broiler chicken [127]. Both AMR and MDR isolates of *E. coli*, *V. cholerae*, and *Salmonella* spp. were identified in large numbers in the poultry sector in Bangladesh [128,129]. Food items such as chicken nuggets were reported to be contaminated with MDR bacteria in Dhaka, Bangladesh [130]. In large animals, separate studies reported quinolone-resistant *E. coli* in apparently healthy cattle; gatifloxacin-resistant *E. coli* in raw milk of cattle and buffalo; ampicillin, oxytetracycline, tetracycline, and amoxicillin-resistant *P. aeruginosa* from abscesses of cattle; and azithromycin, tetracycline, erythromycin, oxytetracycline, and ertapenem-resistant *E. coli* and *Salmonella* spp. from dairy farms [109,131,132,133].

In India, it has been reported that 70–90% of *Enterobacteriaceae* are ESBL-positive and that the CTX-M-15 β-lactamase is dominating in India following its first detection in Delhi in 2000 [134]. ESBL in animals also rose from 12 to 33% from 2013 to 2019 [135]. A high prevalence of 26% has been reported in north India [136].

In Pakistan, ESBL have been frequently reported in community and hospital settings as well as in animals from different parts of the country. The *blaCTX−M* gene has been reported as a predominant genotype in this region, while *blaTEM* and *blaOXA* genes were less common in healthcare settings [100]. In another study, 25.41% of ESBL-producing *E. coli* was detected in milk from mastitis-affected cattle, an alarming percentage for the whole region [115].

## 9. Future Threats of ESBL in South Asian Developing Countries

Undoubtedly, infections caused by ESBL-producing organisms are of great concern to the medical world. The rising prevalence rates along with the dire lack of effective antimicrobial therapy are alarming. Carbapenem is the drug of choice for the treatment of infection caused by ESBL-producing enterobacteria. However, carbapenem-resistant *Enterobacteriaceae* are superbugs that can cause significant morbidity and mortality [137]. New Delhi metallo-β-lactamase (NDM) can inactivate carbapenem and other β-lactam antibiotics except aztreonam [138]. The NDM variant might have evolved in *Enterobacteriaceae*, *Vibrionaceae*, and other non-fermenters by single and double amino acid residue substitutions at different positions [139]. Therapeutic options may be more limited as a result of the evolution of new variants of NDM [140]. Genome transfer among unrelated bacterial species is not the only factor responsible for the increase and spread of NDM variants worldwide. Human factors, such as travel, sanitation, and food production and processing, can also amplify the issue [141]. NDM-17 and NDM-20 were reported in ST1114 *E. coli* isolated from chicken and pig, respectively, in China, indicating that food animals have become a reservoir of NDM-producing bacteria [142]. For the treatment of infections caused by NDM producers, the last resort antibiotic colistin is commonly used. However, a colistin-resistant mcr-1 gene in *E. coli* was recently detected from a pig farm in China [143,144]. From 2016 to date, several plasmid-mediated colistin-resistant mcr genes have been detected in *E. coli*. The use of colistin has been limited in humans because of nephrotoxicity, but it has been used extensively in the veterinary field for decades for prevention and treatment of enteritis and as a growth promoter [145,146]. Thus, the prevalence of colistin-resistant mcr-1 gene variants in *E. coli* was higher in animals than in humans, indicating that the livestock sector was most likely the main source of colistin resistance amplification and spread in animals and in the human population [147,148].

In Bangladesh, reports indicated the emergence of carbapenem-resistant bacteria harboring *bla*OXA-48, *bla*NDM-1,5 and *bla*VIM-5, and colistin-resistant *K. pneumoniae* harboring *mcr-8* in clinical isolates [149,150]. The MDR NDM-1 was first detected in *Klebsiella pneumoniae* in an individual who traveled to India in 2008 [151]. Since then, NDM-1 has been found in various species of *Enterobacteriaceae*, *Acinetobacter* spp., and *Pseudomonas* spp. and 24 variants of NDM have been identified. Another superbug is the Bengal Bay clone of *Staphylococcus aureus*, which originated from the Indian subcontinent in the 1960s [152]. Additionally, methicillin-resistant *Staphylococcus aureus* (MRSA) remains a current and a future threat to hospital patients [153]. In 2018, an outbreak of extensively drug-resistant (XDR) *Salmonella enterica* serovar *Typhi* was reported in Pakistan and Bangladesh [154]. Poor sanitation and overuse of antibiotics are considered the main culprits for the emergence of superbugs in these regions and are expected to impact the South Asian region in future years.

## 10. Conclusions

Antimicrobial resistance is an ongoing global issue. During the COVID-19 pandemic, a decline in the rising trends of ESBL infections, as compared to rates observed before the pandemic, was observed [155]. Travel restrictions, in addition to overall precautions for preventing the spread of infections, might have contributed to this. This gives us hope that proper antimicrobial stewardship could contribute to the reduction of transmission rates of ESBL infections in the future. This is in spite of studies indicating a higher prevalence of other MDR infections, such as MRSA, vancomycin-resistant *Enterococci* (VRE), carbapenem-resistant *Enterobacteriaceae* (CRE), and carbapenem-resistant *Acinetobacter baumannii* (CRAB), during the COVID-19 pandemic [155]. Given this, detailed molecular studies on ESBL-producing bacteria and other superbugs could help identify changing mechanisms of resistance, transmission routes, and alternative drug targets to control pathogenicity. Moreover, there is an urgent need to develop precise diagnostic tools, new drugs, and novel strategies against difficult-to-treat antibiotic-resistant pathogens, including the use of antibiotics in combination or with adjuvants, bacteriophages, antimicrobial peptides, nanoparticles, antibacterial antibodies, and photodynamic light therapy. A One Health approach of systematic surveillance of ESBL across the public health and animal health sectors could be helpful. Finally, there should be more control of the use and release of antibiotics in the environment in South Asian countries and elsewhere in the world.

## Figures and Tables

**Table 1:** Diagnostic tools for detection of extended-spectrum β-lactamases (ESBL).

Screening Tests			Confirmatory Tests				Rapid Kit Test
			Phenotypic Methods			Genotypic Methods	
Test Name	Antibiotic	Sensitivity	Test Name	Antibiotic	Sensitivity		
Kirby-Bauer disks	Cefotaxime, ceftriaxone, ceftazidime, or aztreonam	92–93%	Double-disk synergy test (DDST)	Cefotaxime, ceftriaxone, ceftazidime, or aztreonam	70–80%	PCR	Cica Beta Test 1/HMRZ-86/Chromogenic cephalosporin
Vitek			Combination disk method,	Cefotaxime and cefepime	100%	Nucleotide sequencing	
			E-test ESBL strip	Cefotaxime and ceftazidime	71–73%	Isoelectric point determination	
				Cefepime	90%	DNA probes	
						Oligotyping method	
						PCR-RFLP	
						PCR-SSCP	

**Table 2:** Current status of ESBL as reported in Bangladesh, India, and Pakistan public health sectors from 2015 to 2023.

	Country	ESBL	*Enterobacteriaceae*	Source	Prevalence	Reference
1	Bangladesh	*bla*_CTX-M-15_	*E. coli*	Urine	80%	[83]
2	India	*bla*_CTX-M-15_	*E. coli*	Skin and soft tissue	70%	[84]
3	India	*bla*_CTX-M-15_	*E. coli*	Urine, pus, extra intestinal clinical samples	25%	[85]
4	Bangladesh	*bla*_TEM_	*E. coli*	Urine	50%	[86]
5	Bangladesh	*bla*_CTX-M-15_	*E. coli*	Rectal swabs	48.2%	[87]
		*bla*_CTX-M-1_			11.1%	
		*bla*_SHV-12_			11.1%	
		*bla*_CTX-M-14_			7.4%	
		*bla*_CTX-M-27_			7.4%	
		*bla*_CTX-M-9_			3.7%	
		*bla*_CTX-M-14b_			3.7%	
		*bla*_SHV-28_			3.7%	
		*bla*_TEM-12_			3.7%	
6	Bangladesh	*bla*_CTX-M-1_	*E. coli*	Clinical specimens	33.9%	[88]
		*bla*_CTX-M-1_	*K. pneumoniae*		51.4%	
7	Bangladesh	Non-specific	*E. coli*	Urine	25.84%	[81]
		Non-specific	*Klebsiella pneumoniae*		6.6%	
8	Bangladesh	*bla*_TEM_	*E. coli*	Urine	22.7%	[89]
		*bla*_CTX-M_			24.2%	
		*bla*_SHV_			4.3%	
9	Bangladesh	Non-specific	*K. pneumoniae*	Tracheal swabs, sputum, wound swabs, pus, blood, urine	50%	[90]
		Non-specific	*K. oxytoca*		25%	
10	Bangladesh	*bla*_CTX-M-3_	*Pseudomonas* spp.	Urine, swab, pus	78.0%	[91]
		*bla*_CTX-M- 14_			80.0%	
11	Bangladesh	*bla*_TEM_	*E. coli*	Stool	41%	[62]
		*bla*_CTX–M–group–1_			96%	
12	India	*bla*_CTX-M-15_	*E. coli*	Urine	52%	[92]
		*bla*_OXA-2_			8%	
13	India	Non-specific	*E. coli*	Pus	9.8%	[93]
				Urine	82.6%	
14	North-East India	*bla*_CTX-M_	*E. coli*	Urine, sputum, vaginal discharge	54.34%	[94]
		*bla*_TEM_			60.86	
		*bla*_SHV_			63.04%	
15	South India	*bla*_CTX-M-15_	*E. coli*	Urine, wound swab, sputum, pus, endotracheal secretions, bronchoalveolar lavage, bile fluid	90%	[95]
16	Bihar, India	*bla*_TEM_	*E. coli*	Stool	51.8%	[96]
		*bla*_SHV_			68%	
		*bla*_CTX-M_			86.1%	
17	India	*bla*_SHV_	*Pseudomonas aeruginosa*	Urine, blood, sputum, endotracheal aspirate	15.1%	[97]
		*bla*_TEM_			57.1%	
18	Pakistan	*bla*_CTX-M-15_	*E. coli*	Fecal samples	86.2%	[98]
19	North-West Pakistan	Non-specific	*P. aeruginosa*	Burn patients	35.85%	[99]
20	Lahore, Pakistan	*bla_CTX - M_*	*E. coli, Klebsiella* spp., *Pseudomonas aeruginosa*, *Enterobacter* spp., *Acinetobacter* spp.	Urine, pus, wound swabs	76%	[100]
		*bla_TEM_*			28%	
		*bla_SHV_*			21%	
21	Faisalabad, Pakistan	*bla*_CTX-M-1_	*E. coli*	Dog owners	59%	[101]
				Cat owners	73.9%	
				Veterinary professionals	80%	
22	Pakistan	*bla*_CTX-M1_	*K. pneumoniae*	Hospital waste	71%	[102]
		*bla*_TEM_			53%	
		*bla*_SHV_			6%	
23	Lahore, Pakistan	*bla*_CTX-M-I_	*E. coli*	Clinical specimens	72.1%	[103]
		*bla*_CTX-M-II_			8.5%	
24	Peshawar, Pakistan	*bla*_CTX-M-15_	*Pseudomonas aeruginosa*	Clinical specimens	19.71%	[104]
25	Lahore, Pakistan	Non-specific	*E. coli*	Healthy individuals	57.0%	[105]
				Patients	53.0%	
26	Faisalabad, Pakistan	*bla*_CTXM-1_	*E. coli*	Urine	70%,	[106]
		*bla*_TEM-1_			74.4%	
		*bla*_CTXM-15_			49%	

**Table 3:** Current status of ESBL as reported in Bangladesh, India, and Pakistan animal health sectors from 2015 to 2023.

	Country	ESBL	*Enterobacteriaceae*	Species	Source	Prevalence	Reference
1	Bangladesh	*bla*_TEM_	*E. coli*	Chicken	Droppings	78%	[86]
2	India	*bla*_CTX-M-15_	*E. coli*	Poultry	Meat	17%	[107]
3	Pakistan	*bla*_CTX-M-15_	*E. coli*	Migratory birds	Fecal samples	92.3%	[61]
4	Bangladesh	*bla*_TEM_	*E. coli*	Chicken	Meat	86%	[108]
5	India	*bla*_CTX-M-15_	*E. coli*	Piglets	Fecal samples	2.94%	[109]
		*bla*_TEM_				6.47%	
6	India	*bla*_CTX-M-1_	*E. coli*	Piglets	Fecal samples	55.55%	[110]
7	West Bengal, India	*bla*_CTX-M_	*Klebsiella* spp.	Broiler	Cloacal swabs	10.7%	[111]
		*bla*_SHV_				51.5%	
		*bla*_TEM_				48.5%	
8	West Bengal, India	*bla*_CTX-M_	*E. coli*	Cattle	Milk	54.54%	[112]
9	Assam and Meghalaya	*bla*_CTX-M_	*E. coli, Salmonella*.	Pigs	Fecal samples	0.67%	[113]
		*bla*_TEM_				2.76%	
10	Faisalabad, Pakistan	*bla*_CTX-M-1_	*E. coli*	Dogs	Fecal samples	81.8%	[101]
				Cats		73.9%	
11	Pakistan	*bla*_CTX-M-15_	*E. coli*	Wild birds	Fecal samples	92.3%	[61]
12	Punjab, Pakistan	*bla*_TEM-1_	*Salmonella enterica* serovar *Infantis*	Poultry	Post mortem specimens	44·4%	[114]
13	Lahore, Pakistan	Non-specific	*E. coli*	Cattle	Feces	66.0%	[105]
				Chicken	Feces	59.0%	
				Cattle, Chicken	Raw meat	70.0%	
14	Pakistan	*bla*_CTX-M-15_	*E. coli*	Cows	Mastitic milk samples	63.04%	[115]
		*bla*_CTX-M-55_, *bla*_CTX-M-14_				8.69%	
		*bla*_CTX-M-3_, *bla*_CTX-M-1_				2.17%	
		*bla*_TEM_				47.82%	
		*bla*_SHV_				17.39%	
15	Pakistan	*bla*_CTX-M_ *bla*_TEM_	*E. coli*	Backyard chicken	Cloacal swabs	45.1%	[116]
16	Bangladesh	*bla*_SHV_	*E. coli*	Broiler	Raw meat swabs	12.8%	[117]
				Layer		7.61%	

## Data Availability

All relevant data are included within the manuscript.
